# Ball Possession Effectiveness in Men’s Elite Floorball According to Quality of Opposition and Game Period

**DOI:** 10.2478/hukin-2013-0062

**Published:** 2013-10-08

**Authors:** Miguel-Ángel Gómez, Miguel Prieto, Javier Pérez, Jaime Sampaio

**Affiliations:** 1 Faculty of Physical Activity and Sport Sciences, Polytechnic University of Madrid, Spain.Research.; 2 Center for Sport Sciences, Health and Human Development, University of Trás-os-Montes e Alto Douro at Vila Real, Portugal.

**Keywords:** notational analysis, situational variables, team sports, performance analysis

## Abstract

The aim of the present study was to identify the importance of floorball tactical variables to predict ball possession effectiveness, when controlling quality of opposition and game periods. The sample was composed by 1500 ball possessions, corresponding to 14 games randomly selected from the International Championships played during 2008 and 2010 (World Championship, Four nations tournament and classificatory phases for World Championship) by teams from different competition levels (HIGH, INTERMEDIATE and LOW). The effects of the predictor variables on successful ball possessions according to the three game contexts (HIGH vs. HIGH; HIGH vs. LOW; LOW vs. LOW games) were analyzed using Binomial Logistic Regressions. The results showed no interaction with the game period. In HIGH vs. HIGH games, quality of opposition showed an association with ball possession effectiveness with ending zone, offensive system, possession duration, height of shooting and defensive pressures previous to the shot. In HIGH vs. LOW games the important factors were the starting zone, possession duration, defensive pressure previous to the last pass and to the shot, technique of shooting and the number players involved in each ball possession. Finally, in LOW vs. LOW games, the results emphasized the importance of starting and ending zones, the number of passes used and the technique of shooting. In conclusion, elite floorball performance is mainly affected by quality of opposition showing different game patterns in each context that should be considered by coaches when preparing practices and competitions.

## Introduction

Performance analysis in team sports has studied the importance of individual and collective performances in different game contexts in order to use the information to prepare training tasks according to competition constraints (Hughes and Bartlett, 2002; Sampaio et al., 2010b). In fact, sports performance is much affected by variables such as quality of opposition, game periods, game location, match status or type of competition (Lago, 2009; Lago and Martín, 2007; Lago-Peñas et al., 2011; Sampaio et al., 2010b; Sampaio et al., 2010c). These effects and interactions have been widely studied in team sports such as basketball (Sampaio et al., 2010a; Sampaio et al, 2010c), football (Lago, 2009; Lago and Martín, 2007; Taylor et al., 2008), volleyball (Marcelino et al., 2011), handball (Rogulj et al., 2004) or rugby (Vaz et al., 2010). Research in hockey sports is available in roller (Kingman and Dyson, 2001), field (Sutherland et al., 2006; Spencer et al., 2005) and ice hockey (Geithner et al., 2006), but none is available in floorball (uni-hockey).

Floorball is an indoor hockey sport originated in Sweden in the 1970s (Pasanen et al., 2008) and its practice grew up quickly in European countries. This is a type of floor hockey played in 20 × 40 m low board indoor courts, where two teams of 6 players (one goalkeeper, two defenders and three forwards) try to score a goal to the opponent. All players carry sticks with the exception of the goalkeeper. This is a fast paced game played during three periods of 20 minutes without rough body contacts (Pasanen et al., 2008a, 2008b). The coaches can make substitutions, and in elite floorball it occurs as often as every minute (Paavilainen, 2007). This fact generates different game strategies, in particular the most used offensive tactic is to open up space by running into different positions, and then create open spaces to pass and shoot. This means that players run continuously trying to create passing and shooting positions, and draw the defensive player out of position (Paavilainen, 2007). As the teams attack from different positions and have a wide variety of playing zones (i.e., direct, from the corner, through the center or in the slot/ goal area), and also use different strategies (fast break and set plays) and techniques (i.e., slap shot, puss, backhand) to score a goal, the performance indicators are of great relevance for ball possession effectiveness in floorball (Paavilainen, 2007).

However, the available research in floorball has only focused its attention on injuries (Leivo et al., 2007; Pasanen et al., 2007; Pasanen et al., 2008), and psychological determinants of performance (Høigaard and Ingvaldsen, 2006). There is no research focused on analyzing floorball performance indicators and their influence on team’s performance outcomes. Therefore, the aim of the present study was to identify the tactical variables related to men’s floorball ball possession effectiveness, as well as to control the interactive effect of situational variables (quality of opposition and game periods). The knowledge of these results can provide additional information to be used by coaches in strategical and conditioning planning as well as in the long-term athletic development process.

## Material and Methods

### Sample

The sample was composed of 1500 ball possessions, corresponding to 14 games randomly selected from the International Floorball Championships played during 2008 and 2010 (World Championships, Four nations tournament and classificatory phases for World Championships). The games were provided by the Spanish Floorball-Unihockey Association (SFUF) and by the Swedish Floorball Federation (SFF) being randomly selected from those available on public TV.

### Procedures

The 14 games were analysed through notational analysis performed by four expert technicians. They were all graduates of Sports Sciences with a minimum of 5 years of experience as floorball coaches and were specifically trained for this task. After a 3-week period, to prevent the learning effect, each team re-analysed one game randomly selected. Weighted Kappa correlation coefficients were calculated to assess inter-observer and intra-observer reliability (O’Donoghue, 2010; Robinson and O’Donoghue, 2007). Obtained intra-observer reliability values ranged between 0.81–0.94 and inter-observer values ranged between 0.80–0.90. These values are interpreted as very good/good reliability (Altman, 1991).

### Variables

The ball possession effectiveness was transformed in a dichotomous variable: successful ball possessions (when the offensive team made a shot on the goal or scored a goal), and unsuccessful ball possessions (when the offensive team missed the shot or shot off goal, received an interception of the shot, committed a foul, made a turnover or made any other rule violation).

**Figure 1:**
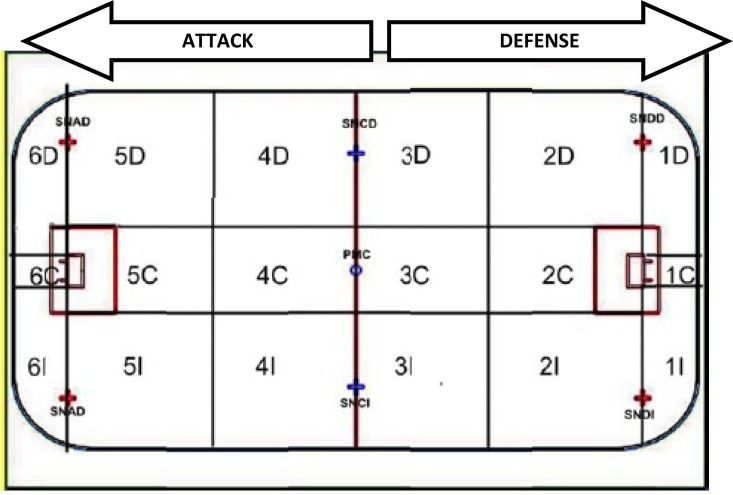
Floorball court zones used in relation to playing tactics (Prieto and Pérez, 2010).

In the absence of any previous research, the variables to analyse performance were determined by 5 expert coaches and researchers (all with a minimum of 5-years of experience as national team coaches and 10-years as youth coaches). The independent variables were related to the zone, task and players’ position. The zone was studied by the starting possession and ending possession in the areas of the court. Eighteen floorball court zones were established previously by Prieto and Pérez (2010) (Figure 1).

A cluster analysis of *k*-means was developed to group the quantitative variables into ranges of values (number of passes, number of players involved and ball possession duration). Then, the task related variables included (i) the number of passes used by each team during ball possession (0 to 4 passes, 5 to 9 passes, 10 to 16 passes, or more than 17 passes used); (ii) the number of players involved in the ball possession (1, 2, 3, 4 or 5 participants); (iii) the offensive systems used by the team (set plays or counter attack); (iv) ball possession duration (0 to 11 seconds, 11 to 30 seconds, more than 30 seconds); (v) DPPS: defensive pressure when the player shoots (HIGH: the defensive player is close to the offensive player with an intense defensive pressure, intermediate (INT): the defensive player keeps a reduced distance trying to avoid any shooting trajectory; LOW defensive pressure: the offensive player is free of any defensive player when making a shot; or no defensive pressure when the offensive player makes a shot without defender and goalkeeper); (vi) DPPP: the defensive pressure when the player passes the previous pass for a shot (HIGH: the defensive player is close to the offensive player with an intense defensive pressure, intermediate (INT): the defensive player keeps a reduced distance trying to avoid any passing trajectory; LOW defensive pressure: the defensive player is far from the offensive player when he made the shot); (vii) trajectory of the last pass (to the right, left, diagonally, or straight forward); (viii) height of the last pass previous to end the attack (high, medium, and low); (ix) technique used to perform the shot (pushing the ball, using the backhand side of the stick, under the player’s knee, or below the player’s knee); (x) height of the shot (high, medium, and low). The players’ positions on the court were either forward or defender.

In order to control the situational variables effects, the game period (first, second and third) was introduced in the models as a covariate. Quality of opposition, defined as the differences in the final (championship) ranking between opposing teams (Gómez et al., 2013), was measured by calculating the competitive level. A two-steps cluster analysis (distance measure: Likelihood; clustering criterion: Schwarz’s Bayesian criterion) was used to group the teams into three competitive levels using the final ranking data provided by the International Floorball Federation (Table 1). The first cluster was high quality teams (HIGH) (with rankings ranged from 1^st^ to 7^th^ positions), the second cluster was intermediate quality teams (INT) (with ranking positions ranged from 8^th^ to 14^th^ positions), and the third cluster was low quality teams (LOW) (rankings lower than 15^th^ place). In order to analyse the influence of quality of opposition (Marcelino et al., 2011) the sample was divided into three groups of game contexts “HIGH vs. HIGH” (*n*= 729 ball possessions), “HIGH vs. LOW” (*n*= 194 ball possessions), and “LOW vs. LOW” (*n*= 527 ball possessions).

### Statistical analysis

Binomial Logistic Regression was used to estimate regression weights and odds ratios of the relation between performance indicators and covariates according to ball possessions effectiveness (Bar-Eli et al., 2006; Marcelino et al., 2011). In this non-linear model of regression, the estimated regression coefficients represent the estimated change in the log-odds, corresponding to a unit change in the corresponding explanatory variable conditional on the other explanatory variables remaining constant (Landau and Everitt, 2004). In the first stage, the performance indicators were tested individually and, in a second stage, the adjusted model was performed with all variables that showed a relation to ball possession effectiveness in the previous stage (Landau and Everitt, 2004). Odds ratios (OR) and their 95% confidence intervals (CI) were calculated and adjusted for ball possession effectiveness. The statistical analyses were performed using SPSS for Windows, version 17.0 (SPSS Inc., Chicago IL), and statistical significance was set at *p*<0.05.

## Results

The distribution of relative frequencies from the studied variables across quality of opposition contexts is shown in Table 2.

In the first stage, the models of the Binomial Logistic Regression were computed with one variable at each step (Table 3), the results showed that during the three game contexts there were no significant interactions with the covariate game period (*p*>0.05). The relationships found reflected the importance in ball possession effectiveness of some tactical variables in each game context that were fitted in the second stage of the model.

The adjusted model (Table 3) fitted the three game contexts (HIGH vs. HIGH: LRT=154.7, *p*<0.0001; HIGH vs. LOW: LRT=104.5, *p*<0.0001; LOW vs. LOW: LRT=95.1, *p*<0.0001).

The results showed no similar relationships in all three contexts, some pairs of interactions were found with a relation between ball possession effectiveness and ending zone (LRT=43.0, *p*=0.0001, and LRT=31.0, *p*=0.0001, respectively) in HIGH vs. HIGH and LOW vs. LOW games. In HIGH vs. HIGH games and HIGH vs. LOW games there was a significant relationship with defensive pressure previous to the final shot (LRT=46.1, *p*=0.0001, and LRT=29.4, *p*=0.0001, respectively). Also, in HIGH vs. LOW games and LOW vs. LOW games there was a significant relationship with starting zone (LRT=31.1, *p*=0.013, and LRT=35.1, *p*=0.006, respectively) and technique of shooting (LRT=9.6, *p*=0.023, and LRT=12.6, *p*=0.005, respectively). On the other hand, some variables were found as significant in isolated contexts, in HIGH vs. HIGH games there were relations with offensive systems (LRT=13.2, *p*=0.0001) and the height of shooting (LRT=7.9, *p*=0.019). In HIGH vs. LOW games there were relations with duration (LRT=8.64, *p*=0.0001), defensive pressure previous to the final pass (LRT=17.2, *p*=0.0001) and players involved (LRT=12.1, *p*=0.002). Finally, in LOW vs. LOW games there were relations with passes used (LRT=12.6, *p*=0.029).

During the HIGH vs. HIGH games, results obtained (Table 4) showed the highest ball possession effectiveness when they ended their attacks in zones 3C (OR=1.90), 3D (OR=4.89), 4C (OR=2.89), 4D (OR=2.83), 4I (OR=2.62), 5C (OR=6.84) and 5D (OR=1.65), when they used set plays (OR=2.19), time durations ranges between 0 and 11 seconds (OR=1.76), when the defensive pressure previous to the final shot was medium (OR=6.54) and when they made a shot with a high height (OR=2.23). In the HIGH vs. LOW games (Table 3) the results obtained showed the highest ball possession effectiveness when they started their attacks in zones 1I (OR=11.67), 3I (OR=14.53), 5D (OR=24.32) and 6D (OR=210.7), when they used time durations ranging between 11 and 30 seconds (OR=33.02), when they used 3 participants in their attacks (OR=11.70) and when the defensive pressure previous to the final shot was medium (OR=4.62) or high (OR=2.66). However, when the teams used the techniques of shooting of backhand (OR=0.04) and pushing the ball (OR=0.30) to end the ball possession, as well as when the defensive pressure previous to the final pass was high (OR=0.10) or intermediate (OR=0.07) they reduced ball possession effectiveness.

Finally, during LOW vs. LOW games (Table 4), the results obtained showed the highest ball possession effectiveness when they ended their attacks in zones 3C (OR=8.68), 4C (OR=2.07), 4D (OR=9.67), 4I (OR=8.95), 5C (OR=4.51), and 5D (OR=9.85), and when they used ball possessions that involved from 4 to 9 passes (OR=13.10). Conversely, when the teams started their attacks on defensive half-court zones 1D (OR=0.11), 2I (OR=0.25), 4C (OR=0.30), 3D (OR=0.26), and when the technique of shooting to end the attack was under the knee (OR=0.52) they reduced the ball possession effectiveness.

**Figure 2:**
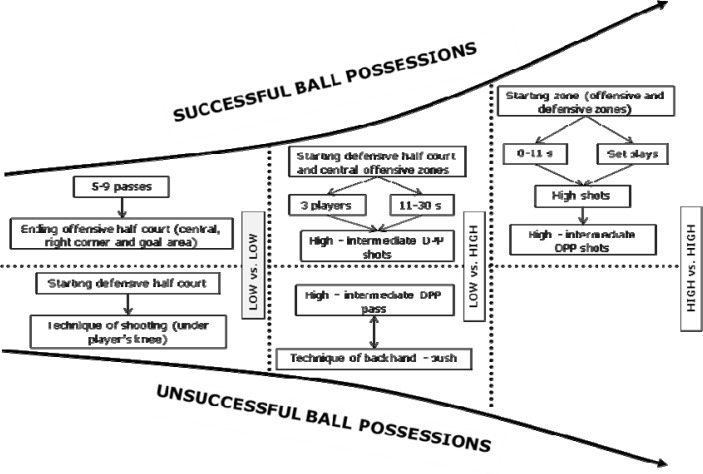
Significant performance indicators related to ball possession effectiveness (successful and unsuccessful) according to quality of opposition.

The Figure 2 explains in a graphical interpretation the relationships between performance indicators and ball possession effectiveness according to quality of opposition (HIGH vs. HIGH; HIGH vs. LOW; LOW vs. LOW).

## Discussion

The aim of the present study was to identify the tactical variables related to men’s floorball ball possession effectiveness, as well as to control the interactive effect of situational variables (quality of opposition and game periods). As was argued, the influence of situational variables may affect the game constraints during floorball games, however, the results suggest a main influence of quality of opposition as found in football (Lago, 2009; Lago and Martín, 2007) and volleyball (Marcelino et al., 2011). In addition, the identified trends were similar across game periods, which contrasts with results obtained in basketball (Sampaio et al., 2010b, c) or volleyball (Marcelino et al., 2011).

In the current study, the different qualities of opposition exhibited different game profiles and allowed to identify different game tactics associated to ball possession effectiveness. These results describe specific performance indicators related to each game context. When two high level teams play (HIGH vs. HIGH) the use of intermediate defensive pressure previous to the shot has an important effect on ball possession effectiveness. In particular, the best players have better technical and tactical knowledge that allows to solve this specific constraint with intermediate defensive pressure. In this way, the best teams use more set plays that increase the chance to obtain a successful ball possession. This fact is related to the different zones of the court used to end the possessions, where the best teams can solve with different offensive strategies that allow ending the attacks in a wide range of zones of the court. Therefore, this game style is less predictable and may generate more defensive actions (Paavilainen, 2007). On the other hand, the possessions ranging between 0 and 11 seconds increased the chance to obtain successful ball possessions. This result is related to the fastbreak situations or transitions that generate more space for each player, and then the space-tempo factors allow the best players to end the tactical situations with higher effectiveness, in accordance with results obtained in basketball (Remmert, 2003). As found in elite field hockey, the goals are scored significantly faster if the ball was recovered in attacking zones, regaining ball possession up the field and increasing the chances for a fast goal (Sunderland et al., 2006).

The technique of shooting from higher heights allowed increasing the chance of obtaining a successful ball possession. In particularly, the best players exhibited excellent technical abilities in its use. Also, the goalkeepers do not carry sticks and can use their legs and hands to stop the ball; therefore, the offensive players have to shoot with higher accuracy when trying to score. In fact, the goalkeeper has a great importance in stops efficiency, in particular Prieto and Pérez (2011) found that elite floorball goalkeepers can stop up to 80% shots per game (Prieto and Pérez, 2011).

In HIGH vs. LOW game, the increased chance to obtain successful ball possessions was related with the use of longer ball possessions with more participants. The best teams use more offensive strategies than lower level teams, and this has consequences in creating more shooting situations (Marcelino et al., 2011). Most of the goals in field-hockey occur from repossession obtained out of the goal area from free hits and interceptions (Sunderland et al., 2006). In fact, the best teams may use defensive pressure in attack half court and worst teams have technical and tactical limitations that generate more turnovers.

The technique of shooting of backhand and push (forehand drive) reduced the chance to obtain a successful ball possession. In particular, the push technique was more common in defensive players, as it needs more time and space and they usually use this form as a long distance shot (Paavilainen, 2007). The use of this type of shot does not increase the effectiveness of possessions during organized set plays, this result may reflect that worst teams try to shoot from long distances with this technique. The most effective techniques to score a goal in field hockey are a hit or a deflection, with a reduced percentage of goals scored from a push shot (Sunderland et al., 2006). Conversely, the backhand technique is a difficult skill that needs a higher level of expertise and practice, and is less used by lower level players.

The differences of team quality also reflected the influence of defensive pressure. The use of high or intermediate defensive pressure previous to the shot increased ball possession effectiveness. As was argued, the best teams are more prepared technically and tactically and they can solve these defensive situations with higher efficacy. The low level teams may show less concentration during ball possessions on the open player or the goal when they have high or intermediate defensive pressures, what reduces the effectiveness of last passes and shots. In fact, when field-hockey players were tested in specific game situations, the high level players obtained better performances and used less time to solve the tests than lower level players (Nair and Bunker, 2002).

There are different zones of the offensive half court that increased the ball possession effectiveness in LOW vs. LOW games. These trends may reflect that lower level teams are less tactically organized, and their attacks presented more turnovers and consequently frequent attack-defense alternations in both teams. In this game context, the use of more passes (ranged from 5 to 9) allows to increase ball possession effectiveness. These trends are similar to those found in basketball (Remmert, 2003), where successful ball possessions involved a wide spectrum of group tactical actions such as more players, passes and screens to end their attacks.

Finally, the use of slap shot (technique of shooting under player’s knee) reduced the ball possession effectiveness in LOW vs. LOW games. This specific technique requires a great domain of the stick and the tactical situation (Paavilainen, 2007). In fact, in hockey sports the slap shot is the fastest technique for projecting the ball (Villaseñor et al., 2006). The ice hockey players use this technique with high frequency during competitions (Lomond et al., 2007). Therefore, players from these lower level teams may generate more mistakes and unsuccessful shots. The available research in floorball found that the slap shot was the less effective shot (Prieto and Pérez, 2011). Then, the coaches are suggested to train this specific technique to improve players’ effectiveness during competitions.

## Conclusions

In conclusion, the results of the present study indicate the importance of quality of opposition in teams’ tactical indicators. The results found showed different game profiles in each game context, at the highest level (HIGH vs. HIGH games) the teams were more organized and presented more offensive tactics and strategies, and reflected a wide range of defensive tactics and shooting techniques. Conversely, at the lowest levels (LOW vs. LOW games) the teams used safer tactics and strategies with longer possession durations and number of passes, also the players showed lower technical abilities during ending actions that generated missed shots and turnovers. Finally, the HIGH vs. LOW games enhanced the contrasting strategies and tactics used by both confronting teams, where the best teams were more tactically disciplined and organized in defensive pressure and offensive strategies. These results allow the coaches for a better understanding of floorball game constraints. The analysis of tactical trends should be considered when preparing floorball training sessions and competitions. Further research is needed to compare the specific game demands in elite floorball games from different perspectives (technical and tactical abilities, game dynamics, physiological or performance analysis).

## Figures and Tables

**Table 1:** International FloorbalL Federation (IFF) rankings based on the two previous World Floorball Championships (retrieved from www.floorball.org; accessed on 01.21.2012).

MEN’S NATIONAL FLOORBALL TEAMS BY IFF RANKING
Sweden Finland Switzerland Czech Republic Norway Latvia Germany Estonia Russia Poland Canada Slovakia Japan Denmark Singapore Hungary Australia Italy USA Korea Serbia Slovenia Austria Spain

**Table 2:** Distribution of relative frequencies from the studied variables across the three game contexts (HIGH vs. HIGH; HIGH vs. LOW; and LOW vs. LOW) in men’s floorball teams.

Performance indicators	**High vs. High**	**High vs. Low**	**Low vs. Low**

	(n=729) (%)	(n=194) (%)	(n=557) (%)
**Efficacy**			
Successful	38.7	40.2	41.9
Unsuccessful	61.3	59.8	58.1
**Space**			
Starting zone			
1C	1.3	0.0	0.1
1D	2.9	4.3	2.9
1I	3.2	6.8	2.7
2C	9.4	8.7	10.2
2D	8.5	5.3	4.3
2I	6.1	4.7	3.8
3C	7.9	9.2	7.2
3D	5.4	3.6	5.8
3I	4.2	6.2	6.5
4C	4.5	4.6	7.4
4D	5.3	8.2	7.6
4I	7.8	7.2	5.8
5C	5.2	5.1	6.9
5D	8.1	6.7	7.7
5I	6.7	8.2	7.4
6C	0.2	2.1	0.5
6D	7.8	4.6	7.2
6I	5.1	4.6	5.1
Ending zone			
1C	0.0	0.0	0.0
1D	0.0	0.0	0.0
1I	0.0	0.0	0.0
2C	0.1	0.0	0.0
2D	0.1	0.0	0.0
2I	0.0	0.0	0.0
3C	1.4	2.2	2.2
3D	0.8	1.5	0.8
3I	0.2	0.0	0.7
4C	11.3	11.4	14.4
4D	9.8	6.2	14.3
4I	8.3	14.9	12.8
5C	24.1	24.2	22.0
5D	22.2	24.2	15.7
5I	21.2	15.4	16.9
6C	0.4	0.0	0.0
6D	0.1	0.0	0.2
6I	0.0	0.0	0.0
**Task**			
Passes used			
0–4 passes	67.5	84.0	86.3
5–9 passes	21.6	12.8	11.3
10–16 passes	9.4	2.6	1.8
>17 passes	1.5	0.7	0.7
Offensive systems			
Set plays	73.2	63.9	64.8
Fastbreaks	26.8	36.1	35.2
Duration (s)			
0–11 s	65.5	75.2	80.9
11–30 s	6.2	3.6	1.3
>30 s	28.3	21.2	17.8
DPP Shots			
High	15.2	21.6	12.5
Medium	46.1	20.1	74.9
Low	36.8	56.7	11.9
No press	1.9	1.6	0.7
DPP Pass			
High	11.3	12.3	4.6
Medium	54.7	47.4	66.0
Low	21.8	24.4	7.7
No pass	12.2	15.9	21.7
Technic of shooting			
Backhand	6.8	6.8	6.9
Push	33.8	28.3	33.8
Below player’s knee	37.8	49.5	42.4
Under player’s knee	21.6	15.4	16.9
Height of shooting			
High	37.0	23.7	25.9
Medium	52.9	62.4	61.7
Low	10.1	13.9	12.4
**Players**			
Ending Player			
Forward	76.4	72.7	76.9
Defender	23.6	27.3	23.1
Players involved			
1	12.2	15.9	21.6
2	23.8	34.0	42.1
3	47.7	39.8	31.7
4	14.8	10.3	4.6
5	1.3	0.0	0.0
**Covariates**			
Game Period			
First	31.4	23.7	30.7
Second	30.5	37.1	34.3
Third	38.1	39.2	35.0

**Table 3:** Model and fit information for the frequency of technical and tactical indicators performed by the teams during the three game contexts according to ball possessions effectiveness in men’s floorball teams

	Chi-Square of Likelihood Ratio		
	HIGH vs. HIGH χ^2^	HIGH vs. LOW χ^2^	LOW vs. LOW χ^2^
Space			
Starting zone	19.7	31.4^*^	30.7^*^
Ending zone	29.4^**^	14.4^*^	31.4^***^
Task			
Passes used	7.5	5.0	9.4^*^
Offensive system	6.0^*^	3.3	1.2
Duration	7.3^*^	7.8^*^	3.1
DPP shots	47.6^***^	23.5^***^	2.8
DPP pass	7.5^*^	15.7^***^	1.9
Technique of shooting	7.5	10.6^*^	13.1^**^
Height of shooting	8.9^*^	0.1	3.4
Players			
Ending player	0.3	0.5	0.1
Players involved	4.2	17.0^***^	0.1
Covariate			
Game Period	0.4	0.5	0.2
**Adjusted model**	154.7^***^	104.5^***^	95.1^***^
Space			
Starting zone		31.1^*^	35.1^**^
Ending zone	43.0^***^	12.9	31.0^***^
Task			
Passes used			9.0^*^
Offensive system	13.2^***^		
Duration	5.7	8.6^*^	
DPP shots	46.1^***^	29.4^***^	
DPP pass	6.5	17.2^***^	
Technique of shooting		9.6^*^	12.6^**^
Height of shooting	7.9^*^		
Players			
Players involved	4.7	12.1^**^	

* *p*<0.05,

** *p*<0.01,

*** *p*<0.001

**Table 4:** Binomial logistic regression: success in ball possessions as a function of technical and tactical indicators used by men’s floorball teams: HIGH vs. HIGH, HIGH vs LOW, and LOW vs. LOW games (reference category: success in ball possession).

Success in ball possessions	OR (95% CI)
**HIGH vs. HIGH games**	
Ending zone	
3C	1.90 (4.30–8.38)^***^
3D	4.89 (5.39–9.43) ^***^
4C	2.89 (1.51–5.52)^***^
4D	2.83 (1.44–5.58)^***^
4I	2.62 (1.25–5.52)^***^
5C	6.84 (4.19–11.2)^***^
5D	1.65 (1.01–2.69)^***^
Offensive system	
Set plays	2.19 (1.43–3.35)^**^
Duration	
0–11 seconds	1.76 (1.10–2.84)^*^
DPP shots	
Intermediate pressure	6.54 (1.46–19.2)^*^
Height of shooting	
High	2.23 (1.23–4.02)^**^
**HIGH vs. LOW games**	
Starting zone	
1I	11.67 (1.10–123.92)^*^
3I	14.53 (1.03–204.4)^*^
5D	24.32 (1.21–490.86)^*^
6D	210.76 (6.24–711.6)^**^
Duration	
11–30 seconds	33.02 (2.24–486.7)^*^
DPP shots	
High pressure	2.66 (8.57–8.28)^***^
Intermediate pressure	4.62 (1.37–1.55)^***^
DPP pass	
High pressure	0.10 (0.01–0.15)^**^
Intermediate pressure	0.07 (0.01–0.83)^*^
Technique of shooting	
Backhand	0.04 (0.01–0.41)^**^
Push	0.30 (0.04–0.80)^*^
Players involved	
3	11.70 (2.00–68.4)^**^
**LOW vs. LOW games**	
Starting zone	
1D	0.11 (0.03–0.45)^**^
2I	0.25 (0.07–0.89)^*^
3D	0.30 (0.10–0.88)^*^
4C	0.26 (0.09–0.81)^*^
Ending zone	
3C	8.68 (9.87–70.6)^***^
4C	2.07 (9.94–4.34)^***^
4D	9.67 (4.86–19.26)^***^
4I	8.95 (4.48–17.85)^***^
5C	4.51 (2.41–8.41)^***^
5D	9.85 (5.06–19.16)^***^
Passes used	
5 to 9 passes	13.10 (1.08–159.2)^*^
Technique of shooting	
Under player’s knee	0.52 (0.29–0.94)^*^

* p<0.05,

** p<0.01,

*** p<0.001; OR, odds ratios; CI, confidence intervals
